# Group-Level Selection Increases Cooperation in the Public Goods Game

**DOI:** 10.1371/journal.pone.0157840

**Published:** 2016-08-30

**Authors:** Catherine C. Eckel, Enrique Fatas, Sara Godoy, Rick K. Wilson

**Affiliations:** 1 Department of Economics, Texas A&M University, College Station, Texas, United States of America; 2 School of Economics, University of East Anglia, Norwich, England, Great Britain; 3 EssexLab CBESS, University of East Anglia, Norwich, England, Great Britain; 4 Department of Political Science, Rice University, Houston, Texas, United States of America; Middlesex University, UNITED KINGDOM

## Abstract

When groups compete for resources, some groups will be more successful than others, forcing out less successful groups. Group-level selection is the most extreme form of group competition, where the weaker group ceases to exist, becoming extinct. We implement group-level selection in a controlled laboratory experiment in order to study its impact on human cooperation. The experiment uses variations on the standard linear public goods game. Group-level selection operates through competition for survival: the least successful, lowest-earning groups become extinct, in the sense that they no longer are able to play the game. Additional control treatments include group comparison without extinction, and extinction of the least successful individuals across groups. We find that group-level extinction produces very high contributions to the provision of the public good, while group comparison alone or individual extinction fail to cause higher contributions. Our results provide stark evidence that group-level selection enhances within-group cooperation.

## Introduction

Competition between groups has been implicated as a factor in the development of human cooperation [1, 2]. Darwin famously observed that groups “including many members who … were always ready to aid one another, and to sacrifice themselves for the common good, would be victorious over most other tribes; and this would be natural selection” [3]. Thus intergroup competition increases the value of altruistic behavior within the group. Darwin’s view was greeted with considerable skepticism, with critics arguing that selection takes place at the individual level, and that individuals cannot sacrifice individual fitness in favor of the group [4, 5]. While a group of altruists might outcompete a group of free riders, altruism must first be able to spread through a population for this situation to occur, and this is unlikely given the within-group free rider problem. Recent research, drawing on historical data, argues that conflict was prevalent enough and genetic diversity sufficiently extensive that intergroup conflict could support the development of altruism, especially when conflict threatened the survival of the group itself [6–9]. However it is difficult to directly test the impact of intergroup competition on cooperation using archival or observational data. The archeological evidence on conflict among hunter-gatherer societies is partial and limited [7], and conflicts among modern societies are too complex to isolate the role of specific behaviors such as cooperation and altruism.

Laboratory experiments are particularly useful for controlling the range of possible strategies, payoffs and communication of information within and among groups. In this study we isolate the effect of group-level selection by imposing a mechanism for group extinction and measuring its impact on within-group cooperation. Two additional experimental conditions rule out alternative hypotheses about our results, including an identical extinction mechanism operating at the individual level, and an extinction-free group comparison treatment.

The tension between individual and group benefits is well captured by public goods (or social dilemma) games. In these settings altruists pay a price for helping the group when they choose to support provision of the public good. The choice for individuals is to allocate resources to their own or to the group welfare. Contributions to the group benefit all members of the group, but at a cost to the individual. These games have been studied extensively in the social sciences to examine the factors that promote or undermine efficiency-enhancing cooperation [10–13]. The public goods game is an ideal vehicle for our experiment.

Importantly, we do not address how group-level selection might arise or operate, nor on the psychological mechanisms that might be involved in its success or failure, but rather we implement group-level selection and directly test its impact on behavior. Others have focused on competition between groups and noted that rivalries are sufficient to boost cooperative behavior to some extent [14–17]. We introduce an extreme form of group competition, where the survival of the lower-earning group is threatened. We implement group-level extinction in the lab by announcing that the lowest-earning group in the first block of the experiment will not be allowed to play the game in the second block, instead earning only their endowment each period.

Our findings demonstrate that group-level extinction produces a remarkable effect on cooperation, resulting in very high contributions to the provision of the public good. We rule out alternative explanations for the high contributions by running two additional control treatments. First, group comparison alone (without extinction) might enhance intergroup competitiveness, and therefore cause higher contributions. Evidence that competitive pressures can affect behavior is pervasive in laboratory experiments [18]. Merely giving subjects information on their relative choices or earnings can boost contributions [19–22]. For this reason we conduct a treatment with group comparison in the form of information about group-level performance, but without extinction. Second, the threat of extinction alone might be responsible for higher cooperation: that is, subjects facing the possibility of extinction may contribute more to their group, even when selection is at the individual rather than the group level. For this reason, we conduct a treatment where the lowest-earning individuals become extinct. Neither factor alone increases contributions substantially relative to the group extinction treatment.

## Materials and Methods

All experiments were carried out in the LINEEX laboratory at the University of Valencia in 2007 and 2008. At the time LINEEX had a blanket exemption from the Ethics Commission at the University of Valencia. The exemption covered all laboratory experiments in which: subject decisions were made over a computerized network; subjects were given random identification numbers; no physiological measures were collected; subjects were privately paid; and no demographic information was collected about subjects. This experiment fell under that exemption. As part of the consenting procedure, subjects read a statement detailing the fact that any information collected during the course of an experiment would be kept strictly anonymous and that a subject could exit the experiment at any time without penalty. This consenting document was displayed at the time subjects voluntarily entered into the subject pool for the LINEEX laboratory. Contacts for the Ethics Committee at Valencia were posted on the website in the event that a subject had any complaints. No personal information was collected for any of the subjects. The only identifier linking subject actions across the data was a randomly assigned identification number. Only behavioral data was collected. No demographic, attitudinal or any other information was collected from subjects.

A total of 168 subjects took part in the experiment conducted at the experimental laboratory at the University of Valencia (LINEEX). Participants were recruited using LINEEX’s own online recruiting software and were undergraduate students (mostly from business and economics) of the University of Valencia. Subjects were randomly assigned to groups of four to play a standard linear public goods game with a marginal per capita return of 0.5. They were told that they would participate in two Blocks of ten rounds each.

All sessions used a common protocol, apart from the details of the games themselves. Upon arrival, subjects were welcomed and randomly seated at the visually separated computer terminals. The experiment was implemented with Z-Tree software [23]. Participants interacted anonymously via computer screens such that subjects did not know which of the other participants were in their group. Subjects were given a written set of instructions (available in S2 File), which the experimenter read aloud. The instructions included a set of control questions (quiz) about how choices translate into earnings and about the composition of groups and sections. Subjects had to answer all the questions correctly before the experiment could continue. Instructions used neutral language (e.g., “extinction” was not mentioned). The total earnings equaled the sum of their total earnings over all 20 periods and were paid privately in cash at the end of the experiment. On average subjects earned 17 Euros. Each experimental session lasted on average 90 minutes.

The experimental design consists of four treatments in a between-subject design. S1 Table and S1 File include a summary of the different experimental treatments. In each treatment there are two blocks, each block consisting of a ten-period public goods game, with subjects randomly assigned to groups of size four at the beginning of the experiment. In Block 1, groups are randomly assigned to one of four possible treatments, while Block 2 is identical across all groups.

In the *Baseline treatment*, individuals participate in a standard public goods game. Given that its only purpose is to serve as a control treatment, we implement two minor variants of the standard public goods game. In *Baseline 20* individuals play the public goods game for 20 rounds (the total number of rounds of Block 1 and 2). In *Baseline 10* they participate in Block 1 for ten rounds and, after a surprise restart [24], in Block 2 for ten rounds. We opted for this dual baseline because some participants in the other three treatments participate in a twenty round experiment (two blocks of ten rounds, with the same groups throughout), while others only in ten (two blocks of ten rounds, but with groups re-matched after the first set). Our analysis demonstrates that every treatment effect holds when using as control either of the two baseline treatments, or both. Additional statistical analysis is given by S1 Fig and S3–S5 Tables.

The second treatment is *Group Extinction*. Here the groups play the public goods game for ten rounds in Block 1, and are then ranked by total earnings at the end of the Block. Subjects are told at the beginning of the experiment that they will find out whether they are in the top 2/3^rds^ or the bottom 1/3^rd^ of the groups at the conclusion of the ten-round block. The lower-ranked groups are dissolved–they become extinct. The remaining groups participate in Block 2. Extinction in this context means that the subjects are no longer allowed to participate in the public goods game, but continue to sit at their computers and receive the same endowment as the other participants each round. To preserve anonymity they were asked to perform a similar task each period with no consequences on earnings.

The third treatment, *Group Comparison* allows us to test whether information about group ranking alone is responsible for any treatment effect from *Group Extinction*. At the end of Block 1, we rank the groups and inform subjects, as in *Group Extinction*, about whether they are in the top 2/3^rds^ or bottom 1/3^rd^ of groups. Subjects are told at the beginning of the experiment that they will receive information about the relative performance of their group at the end of the first block, before moving on to Block 2. There is no extinction: all individuals participate in both blocks of the game.

The fourth treatment, *Individual Extinction*, allows us test the hypothesis that the threat of extinction alone is responsible for any treatment effects of *Group Extinction*. Here we rank individual subjects according to their performance and remove the lowest performing 1/3^rd^ of subjects, across all groups, from the experiment. Subjects are told at the beginning of the experiment that the lowest-performing individuals will be removed from their groups, and the remaining subjects reconstituted into new groups of four for Block 2. Note that, as in the *Group Extinction* treatment, performance determines the fate of participants. The only difference between the two conditions is the selection level: individuals versus groups. As in *Group Extin*ction, subjects who go extinct continue to receive an endowment, but do not play the game. This is the only treatment where the composition of groups changes from the first to the second block.

Most of the elements of the design are common across conditions. Subjects are randomly assigned to groups of four where they remain for ten periods (or 20 in the case of *Baseline20*). Every period each subject is given a new endowment (50 monetary units–MUs) and must decide how to allocate the MUs between a private or to a group account. The private account returns one MU for each invested unit. All MUs placed in the group account pay off .5MU to all members of the group. Thus from the point of view of the individual, the return to each MU invested in the group account is .5MU, but from the point of view of the group, the joint return is 2MU. The social dilemma is such that everyone is better off if all MUs are invested in the group account, but each player has an incentive to free ride.

Subjects also observe the contributions of others in their group for all prior rounds. Each round, the contributions to the public good are ranked from highest to lowest, but that information is not linked to a subject’s identification number to preserve anonymity. Subjects are told nothing about the contributions of the individuals in the other groups.

In most of the public goods literature, the theoretical predictions are based on the standard assumptions of rationality (payoff maximization) and common knowledge of rationality. Under these assumptions, the Nash Equilibrium of the standard game is for all players to contribute zero. Therefore the standard equilibrium prediction for the *Baseline*, *Group Comparison*, and for Block 2 of all treatments, is to contribute zero. In *Individual Extinction*, free riding is still a dominant strategy, as anyone who contributes will enhance the likelihood of his own extinction. In *Group Extinction*, the standard prediction applies to surviving groups in the second block. In equilibrium, earnings are identical to those obtained by non-surviving groups that continue to receive their endowment. Because there is no incentive to survive in equilibrium, the within-group free rider problem still dominates, and the equilibrium prediction is again zero contributions.

Under standard economic theory, then, free riding is always a dominant strategy in all treatments, so that none of these experimental conditions should affect the behavior of individuals (see a similar discussion by [21]). We note that under other assumptions about beliefs, multiple equilibrium can be sustained in all treatments. We did not elicit beliefs in this study, so are unable to test alternative assumptions about beliefs.

## Results

**Fig 1:**
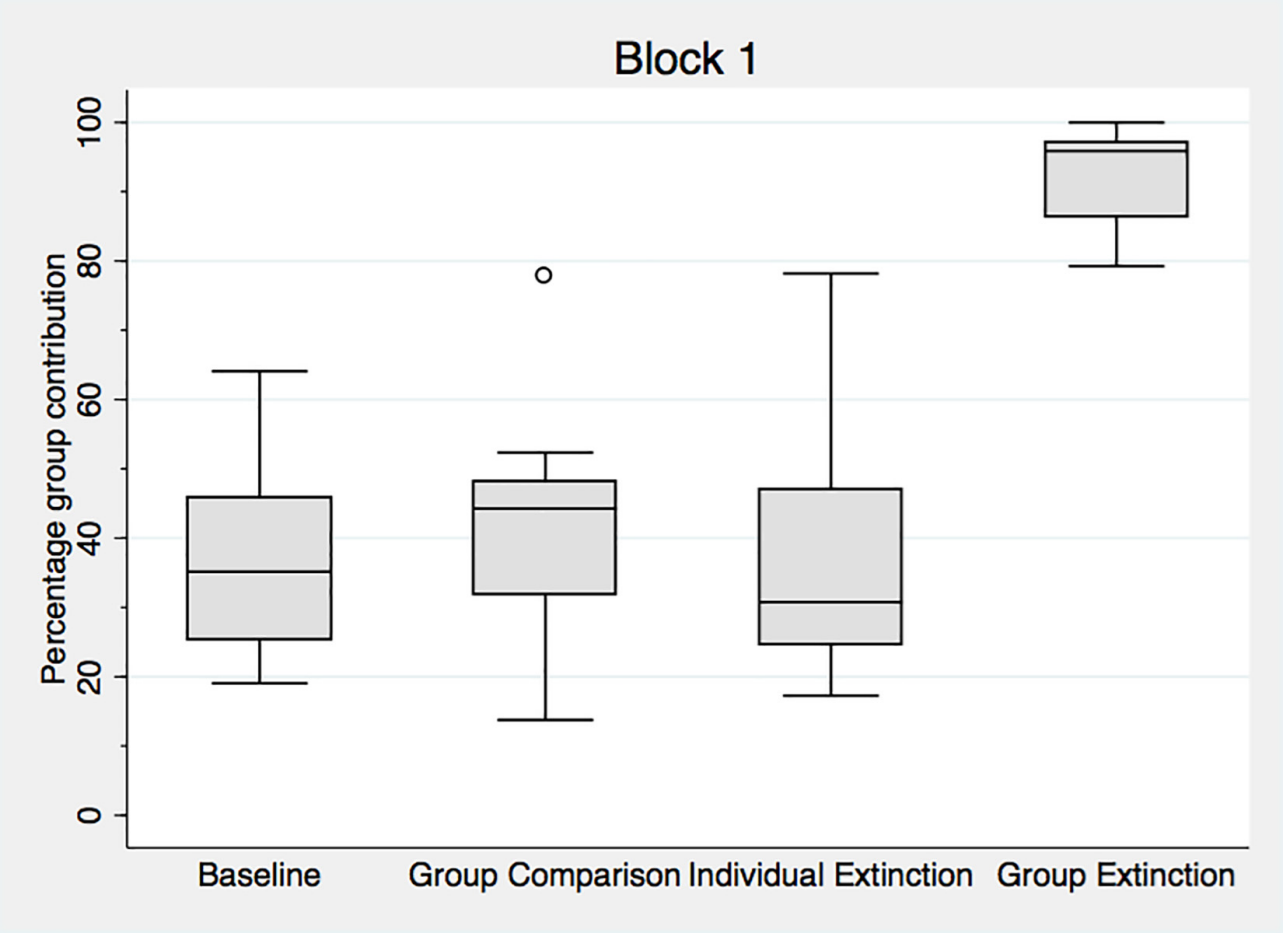
Aggregate Block 1 data for each treatment. Data are the percentage Monetary Units contributed, represented as a box and whiskers plot. The box corresponds to the 25th-75th percentiles, and the line indicates the median value. Whiskers correspond to 1.5 times the interquartile range. Only one value per individual participant (their average contributions) is used in each block. Fig 1 pools the data of both baseline treatments. S5 Fig shows the baseline data disaggregated, while S6 Table shows averages and standard deviations.

Fig 1 presents the distribution of decisions across conditions. The primary finding is that *Group Extinction* leads to greater contributions to the public good (92% of the endowment, on average) than any other treatment (35% in the *Baseline*, 36% in *Individual Extinction*, and 42% in *Group Comparison*). Because average contributions and earnings are intrinsically linked in the game, participants in the *Group Extinction* treatment earn substantially more than those in other treatments. *Group Extinction* leads to a remarkably large and significant contribution increase when compared with the pooled *Baseline* data. S3 Table contains the results of simple non-parametric tests of treatment effects. S4 and S5 Tables provide parametric analysis.

In order to test whether treatment differences are due to relative comparisons, we compare *Group Extinction* and *Group Comparison*. Before making any decisions, subjects in both treatments receive exactly the same instructions concerning the information to be revealed at the end of the first block about the ranking of groups. The difference is that in *Group Extinction* one group out of each three, the one with the lowest performance, no longer participates in the experiment in the second block. The difference is significant (p < .001) with much higher contributions in *Group Extinction*.

In order to test whether the results are due to the threat of extinction alone, we compare *Group Extinction* with *Individual Extinction*. In both treatments one-third of the subjects become extinct by design. The difference is that in *Individual Extinction* it is the lowest-earning subjects across all groups who are removed from the game, while in *Group Extinction* it is the lowest-earning group. The difference is again significant (p-value<0.001). While *Group Extinction* boosts contribution rates, the same is not true for *Individual Extinction* or *Group Comparison*: neither is significantly different from the *Baseline*. See S3 Table for details on the non-parametric tests.

**Fig 2:**
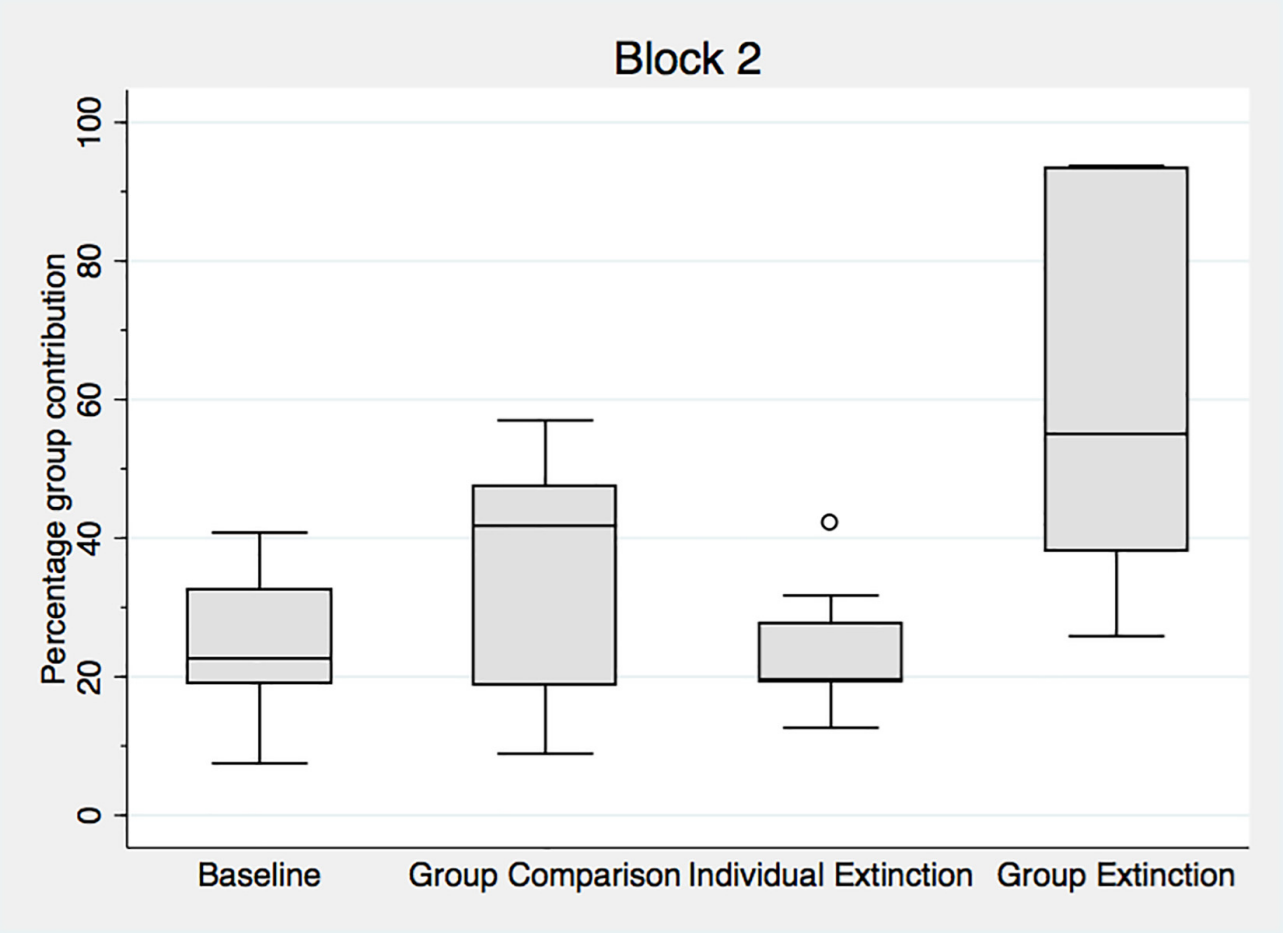
Aggregate data for Block 2 by treatment. Data are the percentage Monetary Units contributed and represented as a box and whiskers plot. The box corresponds to the 25th-75th percentiles, and the line indicates the median value. Whiskers correspond to 1.5 times the interquartile range. One value per individual participant (average contributions across all rounds) is used in each block. See S2 Table for more detail.

Recall that Block 2 is identical across all experimental treatments for the surviving subjects, and consists of ten rounds of a standard public goods game without information or extinction. In this block subjects make decisions in the same public goods game, knowing that the experiment will conclude at the end of the block. Fig 2 presents the distribution of Block 2 contributions to the public good by condition. Comparing outcomes within Block 2, contributions in *Group Extinction* remain well above the other treatments in Block 2 (62% relative to 25% in the *Baseline*, 35% in *Group Comparison* and 23% in *Individual Extinction*), and all pairwise comparisons between *Group Extinction* and the other treatments are significant at least at the 5% level. (The p-values comparing *Group Extinction* to the *Baseline*, *Group Comparison* and *Individual Extinction* treatments are smaller than 0.02, 0.03 and 0.003, respectively).

Comparing Block 1 with Block 2 contributions, (Fig 1 and Fig 2) we observe two main changes. First, while contributions in the three control treatments (the *Baseline*, the *Group Comparison* and the *Individual Extinction*) do not significantly differ across blocks, the decline in the *Group Extinction* condition is significant. A Wilcoxon matched pairs signed rank test shows that differences between Block 1 and Block 2 are significant in the *Group Extinction* condition (p<0.01), but not in the *Group Comparison* treatment (p<0.15) or the *Baseline* with the surprise restart (p<0.25). Not surprisingly, and in line with overwhelming experimental evidence (9) contributions in the last ten rounds of *Baseline20* (with no surprise restart) contributions are lower (p<0.02). The difference between Block 1 and 2 is marginally significant in the *Individual Extinction* condition, using a Mann Whitney rank sum test (this test is used because the composition of groups is not the same).

**Fig 3:**
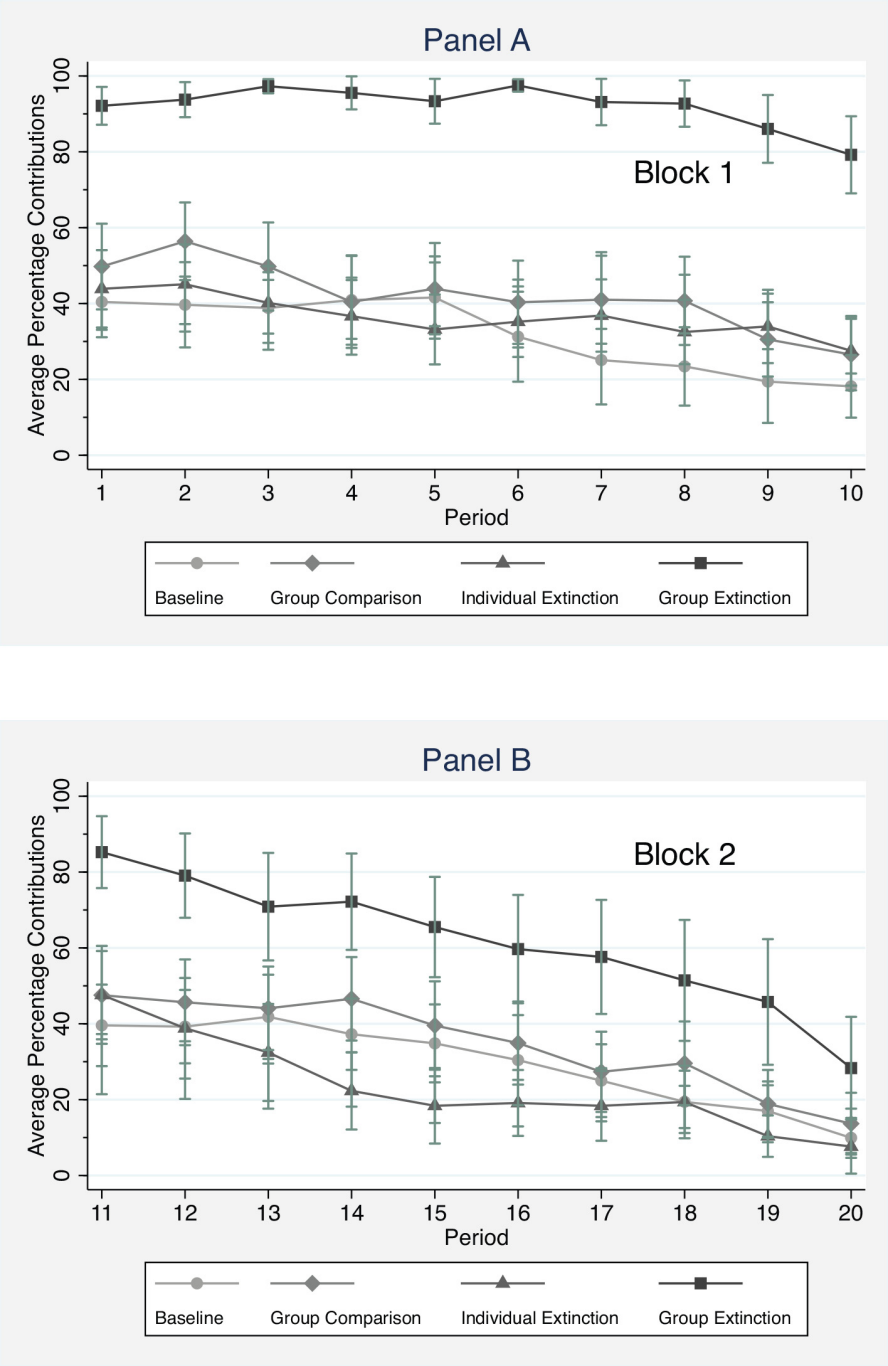
Trends in contributions broken out by treatment. (A) represents the first Block when subjects are being treated. (B) represents the second Block following the treatment condition. Subjects who became “extinct” are excluded from Block 2. Points represent the average individual contributions for that period. 95 percent confidence intervals are plotted for each of the treatments and points. The points are connected to better give a sense of the trends over periods.

Fig 3 presents the average contribution by period in both blocks, across conditions. All treatments have a slight negative trend in contributions towards the end of Block 1 (Fig 1A), but differences between *Group Extinction* and the other three treatments in the initial ten rounds are substantial and statistically significant in any round. Under the threat of group-level extinction the provision of the public good is higher, and remains higher in every period (at above 80% in any period), while in all the other treatments subjects begin by contributing on average just under half of their endowment to the public good and contributions decline, consistent with findings from almost all public goods experiments [11].

All treatments show a “restart effect” with higher contributions in round 11 than round 10, except *Group Extinction*. In the three control treatments subjects demonstrate a positive restart effect, with contributions bouncing back to levels similar to the start of Block 1, a phenomenon commonly observed in public goods games [25]. No restart effect is observed in *Group Extinction*; instead contributions begin close to their levels in Block 1, and decline steadily throughout Block *2*.

We see this post-extinction decline as a valuable robustness check on the long-term effectiveness of *Group Extinction*. Once the selection mechanism is removed, contributions decline faster than in the other treatments. While average contributions remain uniformly higher for the *Group Extinction* treatment compared with the other treatments in Block 2, subjects in this condition decrease their contributions to the public good from 85% to 28% from the beginning to the end of Block 2. Interestingly, no single difference across treatments is statistically significant in round 20 of the experiment.

## Discussion

One of the most important puzzles in the study of human evolution is the prevalence of altruistic behavior in humans. Darwin asserted early on that group competition is an important source of altruism: “When two tribes of primeval man, living in the same country, came into competition, if (other things being equal) the one tribe included a great number of courageous, sympathetic and faithful members, who were always ready to warn each other of danger, to aid and defend each other, this tribe would succeed better and conquer the other (Darwin, 1871, p. 113).”

The concept of group-level selection is both attractive and controversial. Biologists since Darwin have debated whether selection at the group level can support altruism, given the fact that free riders within the group have higher levels of fitness. From the gene’s eye view, altruists can never prevail [4, 26]. These opponents of the group selection argument also assert that there are hardly any instances of group selection, so its effect would necessarily be small. Others argue that group-level selection is so abundant throughout human evolution that it is more than sufficient to explain why humans are altruistic [27]. These and other researchers refer to multi-level selection, with group-level selection one among many possible mechanisms supporting altruistic behavior, and argue that the net effect will depend on the relative strength of the different levels. Furthermore, the idea of cultural selection, developed by anthropologists addressing the same question about the prevalence of altruism, provides a mechanism for acquired group norms of altruistic behavior to be transmitted to future generations without relying on gene-level evolutionary processes [2, 8, 9, 28, 29]. The debate between proponents and opponents focuses on mathematical models and is hampered by limited observational data. Finding clear empirical instances of group-level selection is difficult (but see [30, 31]).

Many open questions remain about the viability and effect of group-level selection as well as the exact mechanism through which it might support altruistic behavior. However, one of its central questions can be addressed in our simple lab experiment. We create an environment where free riding results in higher payoffs within groups, but competition between groups ensures that altruistic groups are more successful. We ask: What is the effect of group-level selection on within-group cooperation in this setting? Our experiment implements an extreme form of group competition in the lab in the form of group extinction: the lowest-earning among three groups playing the public goods game in the lab becomes “extinct,” in the sense that they are no longer allowed to play the public goods game.

Our main finding affirms Darwin’s assertion: Imposing group extinction in the lab results in a dramatic increase in cooperation. The effect is large, even in a setting where the Nash equilibrium of the game is not altered by the extinction mechanism. Control treatments consisting of information-based group comparison and individual-level extinction generate no significant effect relative to a baseline. Group extinction leads to enhanced cooperation as long as the selection mechanism is present; once it is removed, contributions remain higher for a time, but fall quickly towards the Nash equilibrium of zero contributions. The culture of cooperation engendered by the group extinction mechanism has only a brief longer-term carryover after the mechanism is removed.

Of course, other researchers have examined different mechanisms involving group competition to overcome the free rider problem. The mechanisms in these studies include competition for a prize [17, 32], and various inter-group comparisons [22, 33]. Mechanisms not involving group competition, such as sorting of group members [16], or varying forms of communication and sanctioning [13] can also mitigate the free rider problem. In our case, group extinction solves the free rider problem, without communication or sanctions.

Why does *Group Extinction* enhance cooperation? We cannot address whether this change in the environment triggers a psychological mechanism related to group threat [34] or solidifies group identity [35, 36], or perhaps both.

Darwin’s conjecture, that groups may enjoy an advantage whose members are “ready to aid one another, and to sacrifice themselves for the common good,” is affirmed with this study. We show that even when no other cooperation-reinforcing mechanism is present, the threat of group extinction is sufficiently powerful to motivate cooperation and increase within-group cooperation. This result supports the notion that competition between groups is part of what has cultivated human cooperation.

## Supporting Information

S1 Fig Percentage of Group Contributions in the First Block broken out by treatment. Standard error bars are overlaid on the figure.
(TIF)

S2 Fig Percentage of Group Contributions in the Second Block broken out by treatment. Standard error bars are overlaid on the figure.
(TIF)

S3 Fig Distributions of decisions across all 20 periods. The vertical axis indicates the percentage of contributions to the public good. The horizontal axis indicates the amount of the contribution to the public good. Values at the extreme left indicate zero contributions. Values at the extreme right indicate contributing everything.
(TIF)

S4 Fig Distributions of decisions across the First Block (periods 1–10). The vertical axis indicates the percentage of contributions to the public good. The horizontal axis indicates the amount of the contribution to the public good. Values at the extreme left indicate zero contributions. Values at the extreme right indicate contributing everything.
(TIF)

S5 Fig Distributions of decisions across the Second Block (periods 11–20). The vertical axis indicates the percentage of contributions to the public good. The horizontal axis indicates the amount of the contribution to the public good. Values at the extreme left indicate zero contributions. Values at the extreme right indicate contributing everything.
(TIF)

S6 Fig Disaggregated Plots of Baseline data. We use data from 2 Baseline experiments: (i) Baseline with surprise restart (10 + 10 rounds: 6 independent group observations) and (ii) Baseline repeated for 20 rounds (7 independent group observations). Apart from the difference in period 11 we do not find statistically significant differences between them (see S6 Table).
(TIF)

S7 Fig Comparison of Intact Groups Across Block 1 and Block 2. A comparison of the performance of intact groups that played both in Block 1 (horizontal axes) and Block 2 (vertical axes). Any marker above the 45-degree dashed line represents a group that contributes more in the second block than in the first, and the opposite for markers below the line. Not surprisingly, observations from the Group Extinction treatment are all at the right of the diagram (as contributions are high in the first block and lower in the second). However, when we compare performance across conditions in the second block, using the vertical axes, differences vanish. The figure contains information about the performance of surviving groups in the last round (as grey diamonds). In most groups contribution collapses to zero, or gets close to it, and only two groups out of eight still contribute more than 50% of their endowment.
(TIF)

S8 Fig Average contributions by Winners and Losers. Average percentage contributions by individuals are plotted for each period. 95 percent confidence intervals are plotted separately for each treatment and type of individual. The data are for the first Block of data. Ex post we know who remained in the experiment and who did not. We use this information to differentiate between “winners” (who continued in the experiment) and “losers” (who did not continue in the experiment). The lower portion of the figure shows that under the individual extinction (IE) treatment, those who were “winners” normally contributed less than the “losers” in that treatment. However, the differences are not statistically significant. The opposite is the case under the group extinction (GE) treatment. There “winners” contributed somewhat more than “losers” although this difference is not statistically significant. Overall, the behavior of “winners” and “losers” is similar.
(TIF)

S9 Fig Contribution stage. It is the same for each treatment.
(TIF)

S10 Fig Information screen after contribution stage. It is the same for each treatment.
(TIF)

S11 Fig Information screen after the first block for Individual Extinction Low Earner. (TIF)

S12 Fig Information screen after the first block for Individual Extinction High Earner. (TIF)

S13 Fig Information screen after the first block for Group Extinction and Group Comparison Low Earning Groups. In both treatments subjects are given the same information. Only in the Group Extinction treatment do those groups exit the experiment.
(TIF)

S14 Fig Information screen after the first block for Group Extinction and Group Comparison High Earning Groups. In both treatments subjects are given the same information.
(TIF)

S1 File Details about Experimental Design. (PDF)

S2 File Written Instructions for Experiment. (PDF)

S1 Table Summary of Treatments. (PDF)

S2 Table Means (standard deviations) of contributions at the group level. Means are pooled over all periods and then broken out by the first and second blocks.
(PDF)

S3 Table P-values for Wilcoxon Mann-Whitney tests for pairwise treatment differences. The comparisons are for all 20 periods, the first block and the second block. Each comparison includes contributions and earnings information. In the table BSL = Baseline; GC = Group Comparison; IE = Individual Extinction; and GE = Group Extinction.
(PDF)

S4 Table Individual Level Regressions (Panel Random-effects generalized least squares). Analysis is across all periods and then by first and second block. Baseline treatment is the omitted category.
(PDF)

S5 Table Individual Level Regressions (Panel Random-effects generalized least squares). An additional control for the period is included. Baseline treatment is the omitted category.
(PDF)

S6 Table Averages by groups across periods for the baseline treatment. The first part of the table indicates the total average MUs given by the group (out of a possible 200 MUs that could be given in each period). The second part of the table presents Wilcoxon rank-sum tests between the Baseline treatments.
(PDF)
