# Supplementary figures and images for: Group-Level Selection Increases Cooperation in the Public Goods Game
Source: PLoS One. 2016 Aug 30;11(8):e0157840. doi: 10.1371/journal.pone.0157840 (PMC5004815; doi:10.1371/journal.pone.0157840)

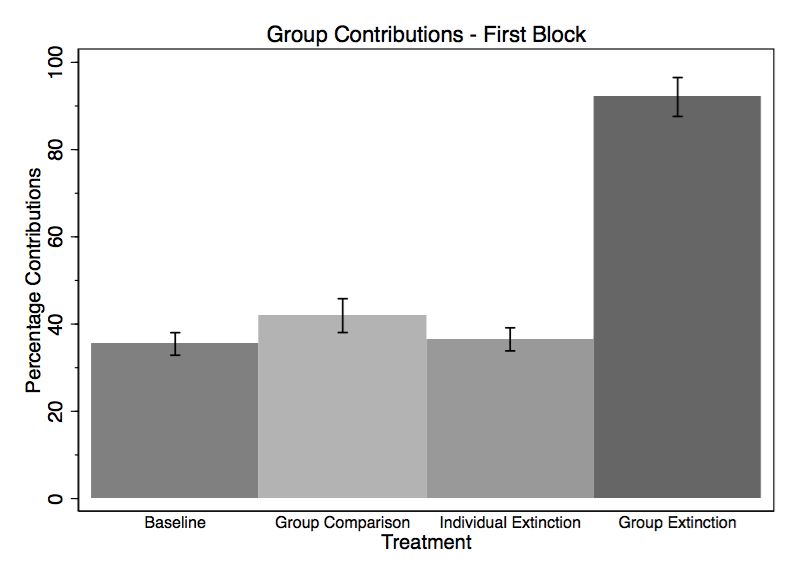

Supplement: S1 Fig — Standard error bars are overlaid on the figure. (TIF) [file pone.0157840.s001.tif]

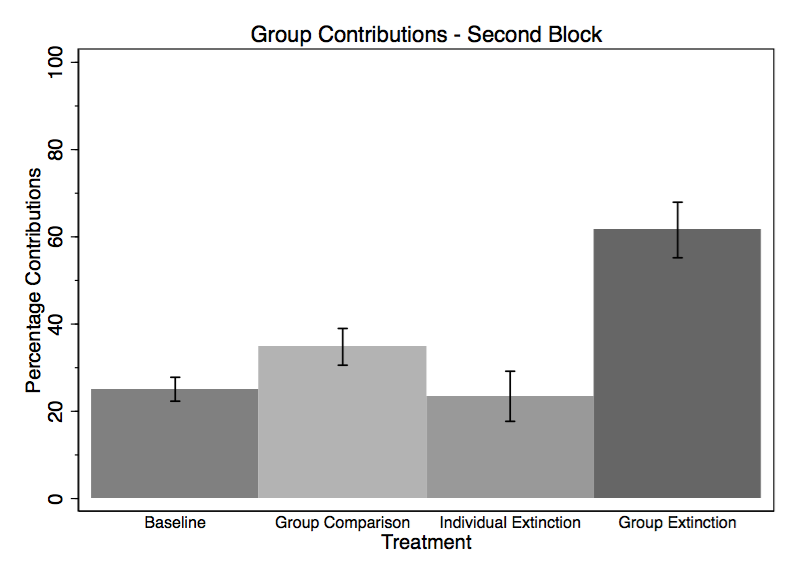

Supplement: S2 Fig — Standard error bars are overlaid on the figure. (TIF) [file pone.0157840.s002.tif]

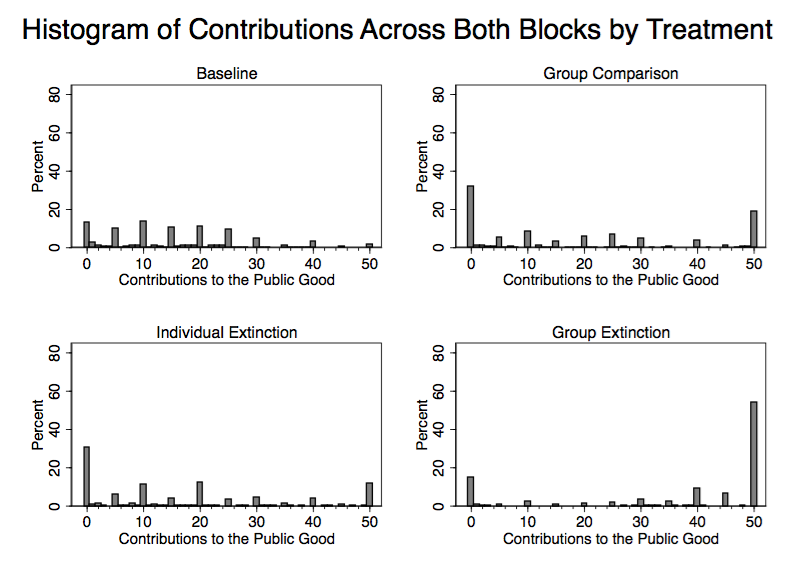

Supplement: S3 Fig — The vertical axis indicates the percentage of contributions to the public good. The horizontal axis indicates the amount of the contribution to the public good. Values at the extreme left indicate zero contributions. Values at the extreme right indicate contributing everything. (TIF) [file pone.0157840.s003.tif]

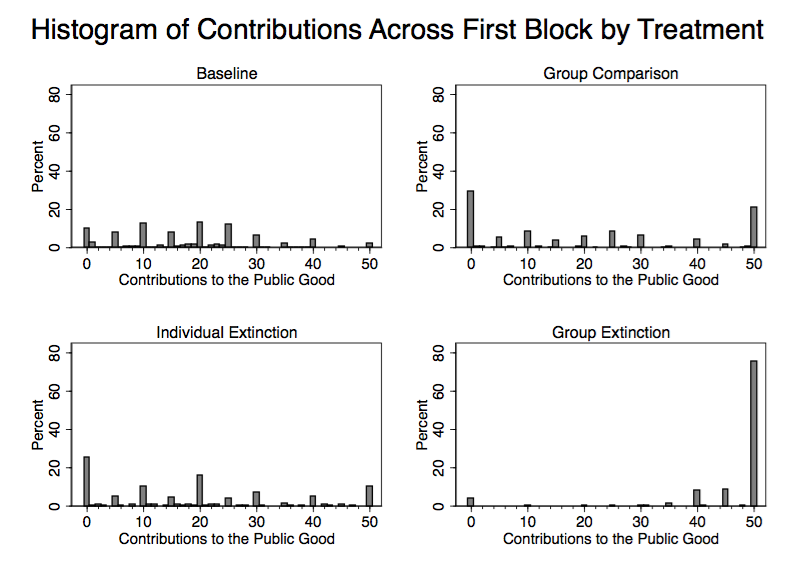

Supplement: S4 Fig — The vertical axis indicates the percentage of contributions to the public good. The horizontal axis indicates the amount of the contribution to the public good. Values at the extreme left indicate zero contributions. Values at the extreme right indicate contributing everything. (TIF) [file pone.0157840.s004.tif]

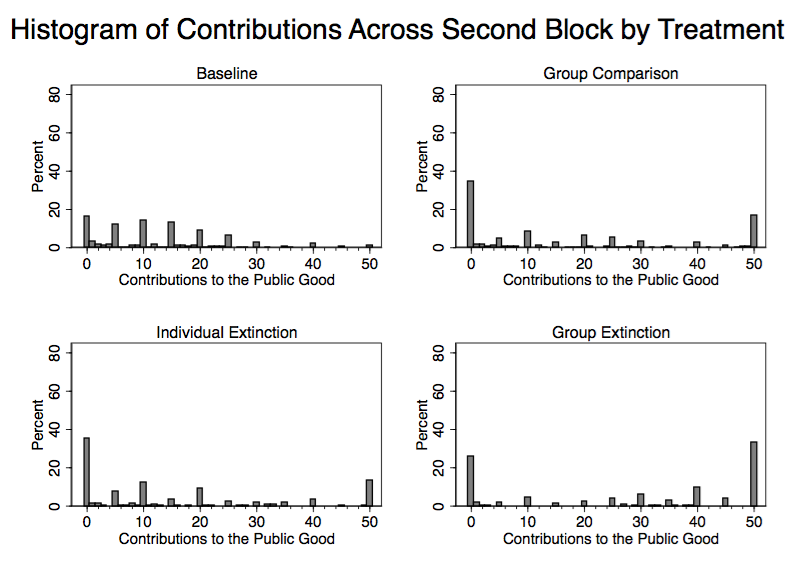

Supplement: S5 Fig — The vertical axis indicates the percentage of contributions to the public good. The horizontal axis indicates the amount of the contribution to the public good. Values at the extreme left indicate zero contributions. Values at the extreme right indicate contributing everything. (TIF) [file pone.0157840.s005.tif]

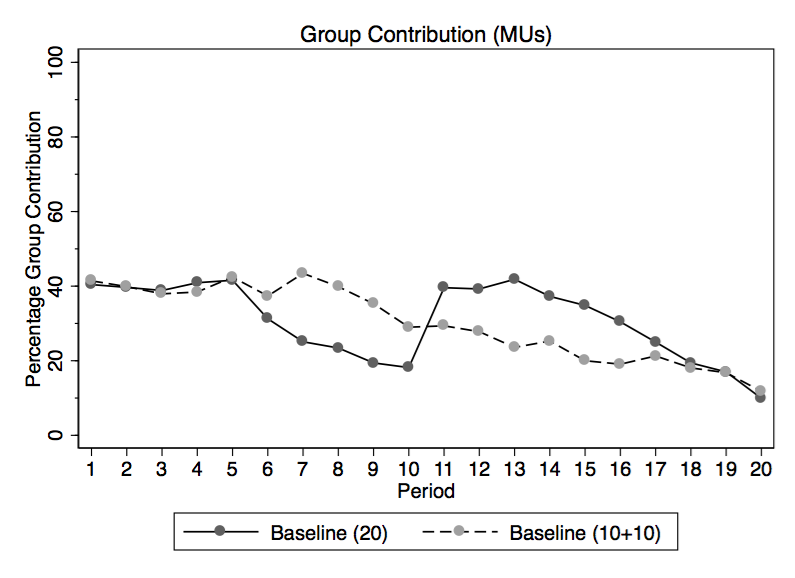

Supplement: S6 Fig — We use data from 2 Baseline experiments: (i) Baseline with surprise restart (10 + 10 rounds: 6 independent group observations) and (ii) Baseline repeated for 20 rounds (7 independent group observations). Apart from the difference in period 11 we do not find statistically significant differences between them (see S6 Table). (TIF) [file pone.0157840.s006.tif]

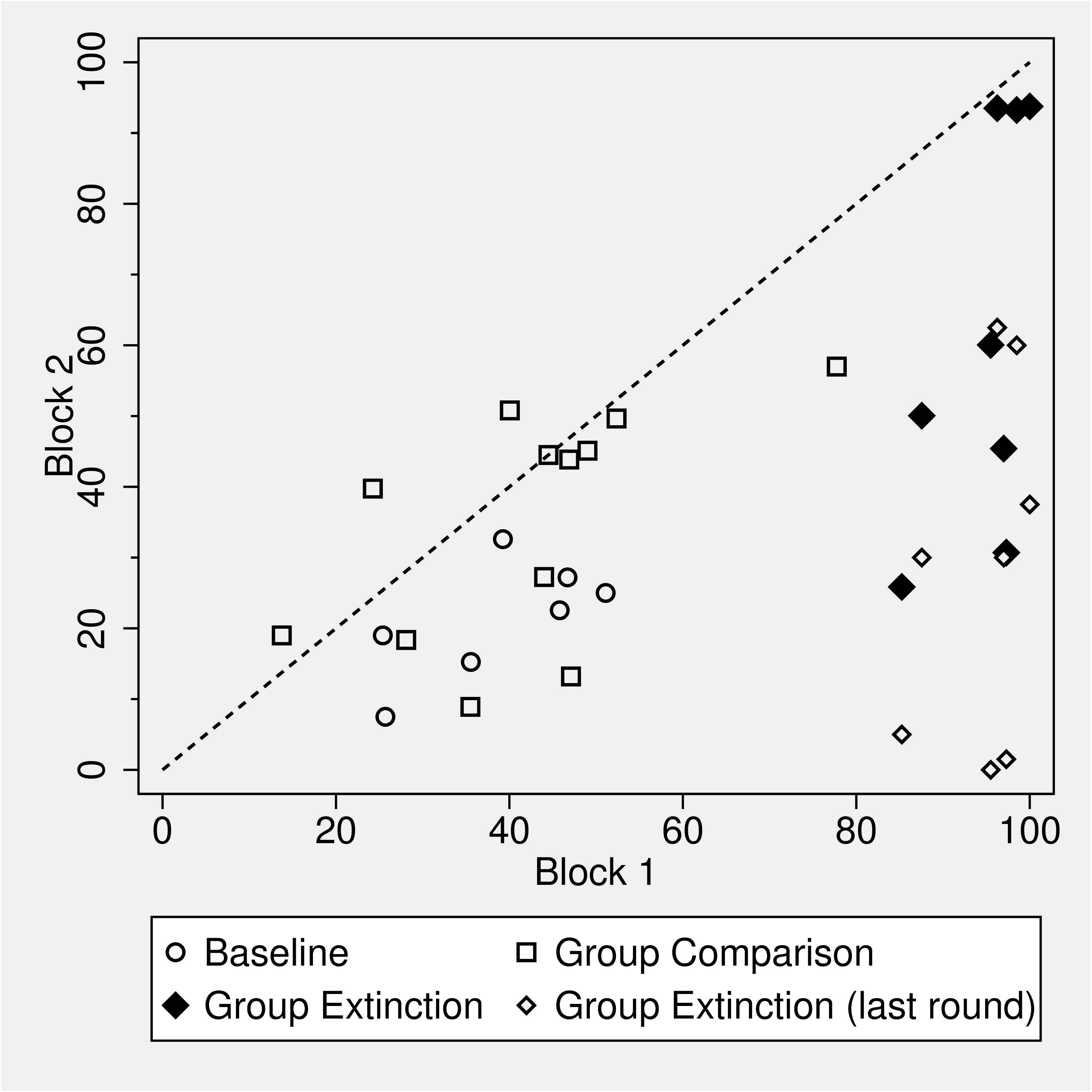

Supplement: S7 Fig — A comparison of the performance of intact groups that played both in Block 1 (horizontal axes) and Block 2 (vertical axes). Any marker above the 45-degree dashed line represents a group that contributes more in the second block than in the first, and the opposite for markers below the line. Not surprisingly, observations from the Group Extinction treatment are all at the right of the diagram (as contributions are high in the first block and lower in the second). However, when we compare performance across conditions in the second block, using the vertical axes, differences vanish. The figure contains information about the performance of surviving groups in the last round (as grey diamonds). In most groups contribution collapses to zero, or gets close to it, and only two groups out of eight still contribute more than 50% of their endowment. (TIF) [file pone.0157840.s007.tif]

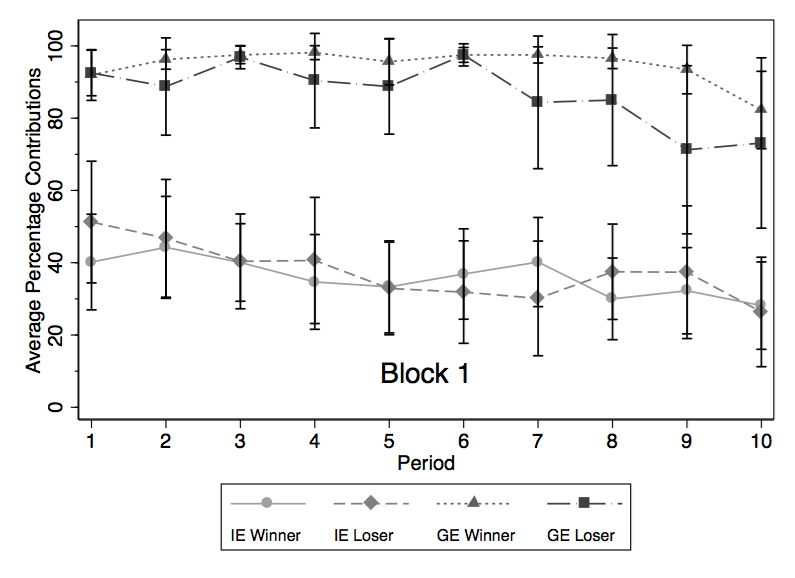

Supplement: S8 Fig — Average percentage contributions by individuals are plotted for each period. 95 percent confidence intervals are plotted separately for each treatment and type of individual. The data are for the first Block of data. Ex post we know who remained in the experiment and who did not. We use this information to differentiate between “winners” (who continued in the experiment) and “losers” (who did not continue in the experiment). The lower portion of the figure shows that under the individual extinction (IE) treatment, those who were “winners” normally contributed less than the “losers” in that treatment. However, the differences are not statistically significant. The opposite is the case under the group extinction (GE) treatment. There “winners” contributed somewhat more than “losers” although this difference is not statistically significant. Overall, the behavior of “winners” and “losers” is similar. (TIF) [file pone.0157840.s008.tif]

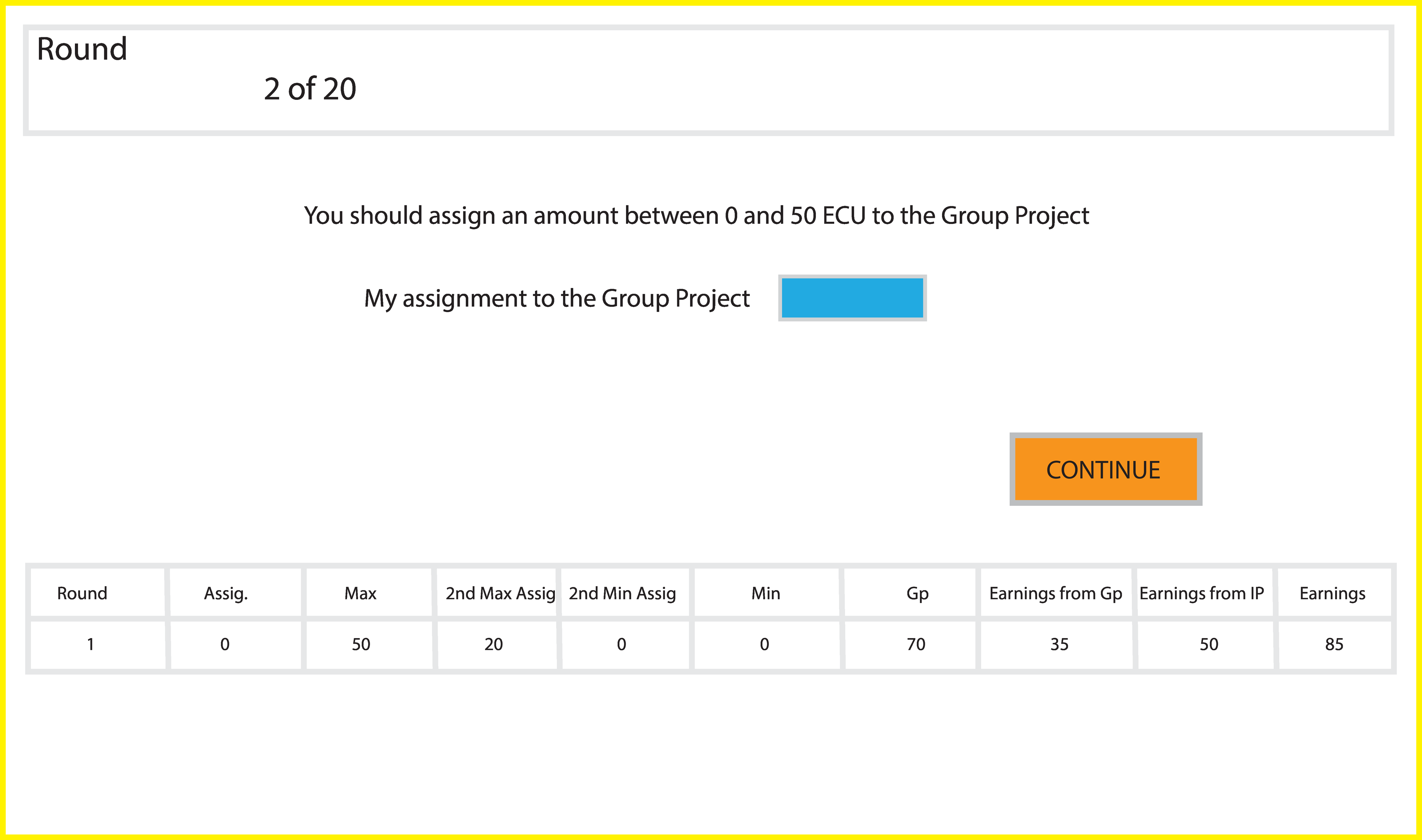

Supplement: S9 Fig — It is the same for each treatment. (TIF) [file pone.0157840.s009.tif]

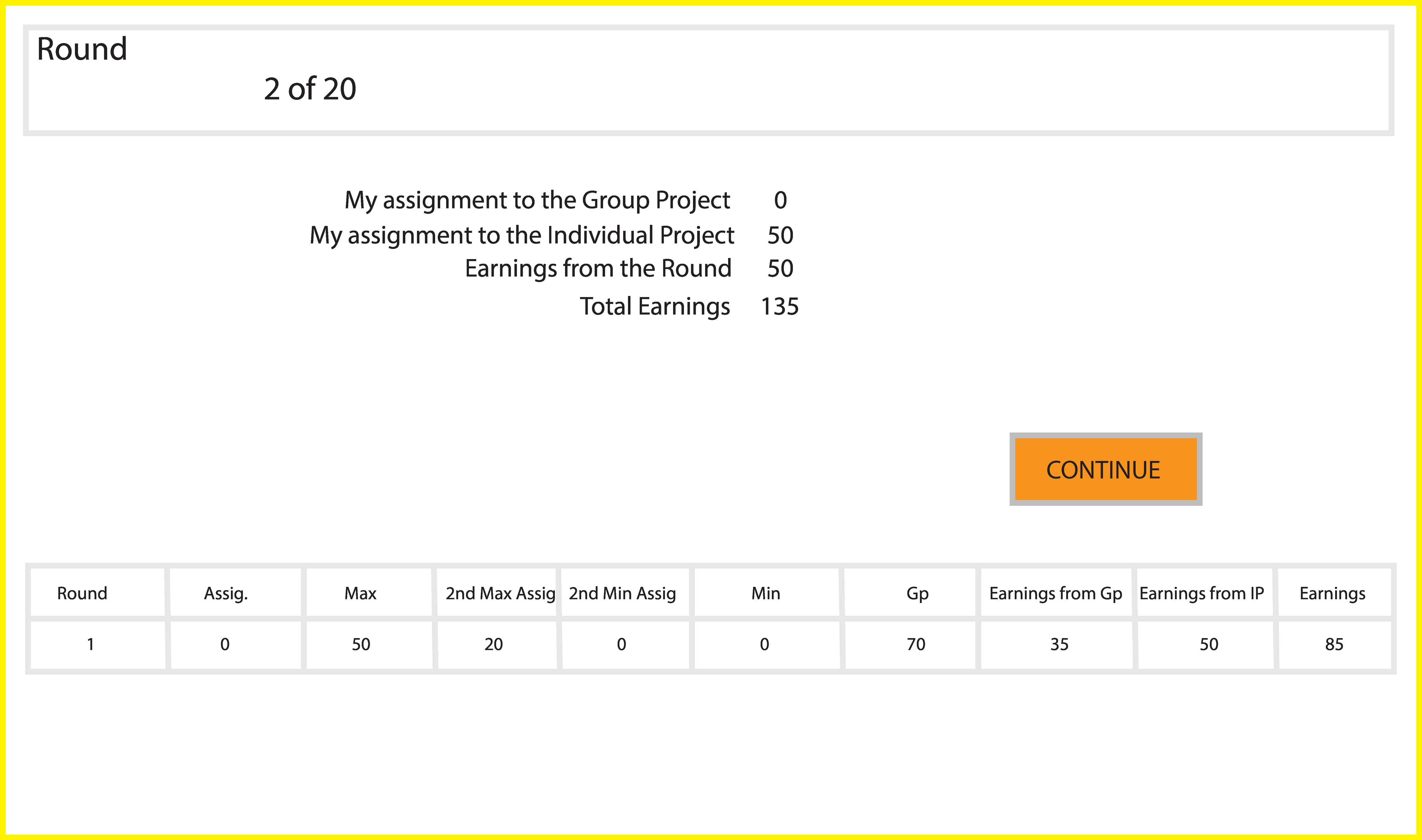

Supplement: S10 Fig — It is the same for each treatment. (TIF) [file pone.0157840.s010.tif]

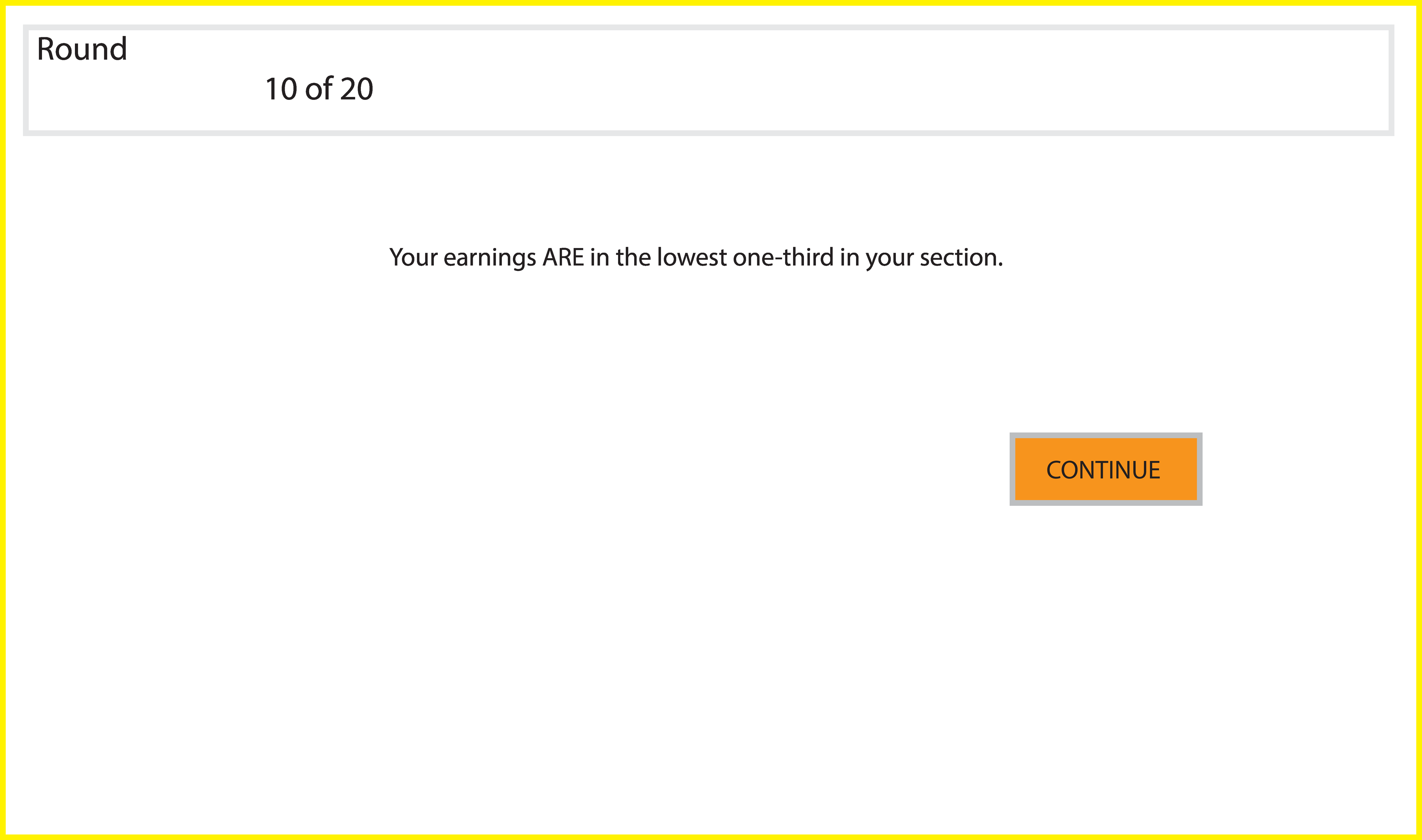

Supplement: S11 Fig — (TIF) [file pone.0157840.s011.tif]

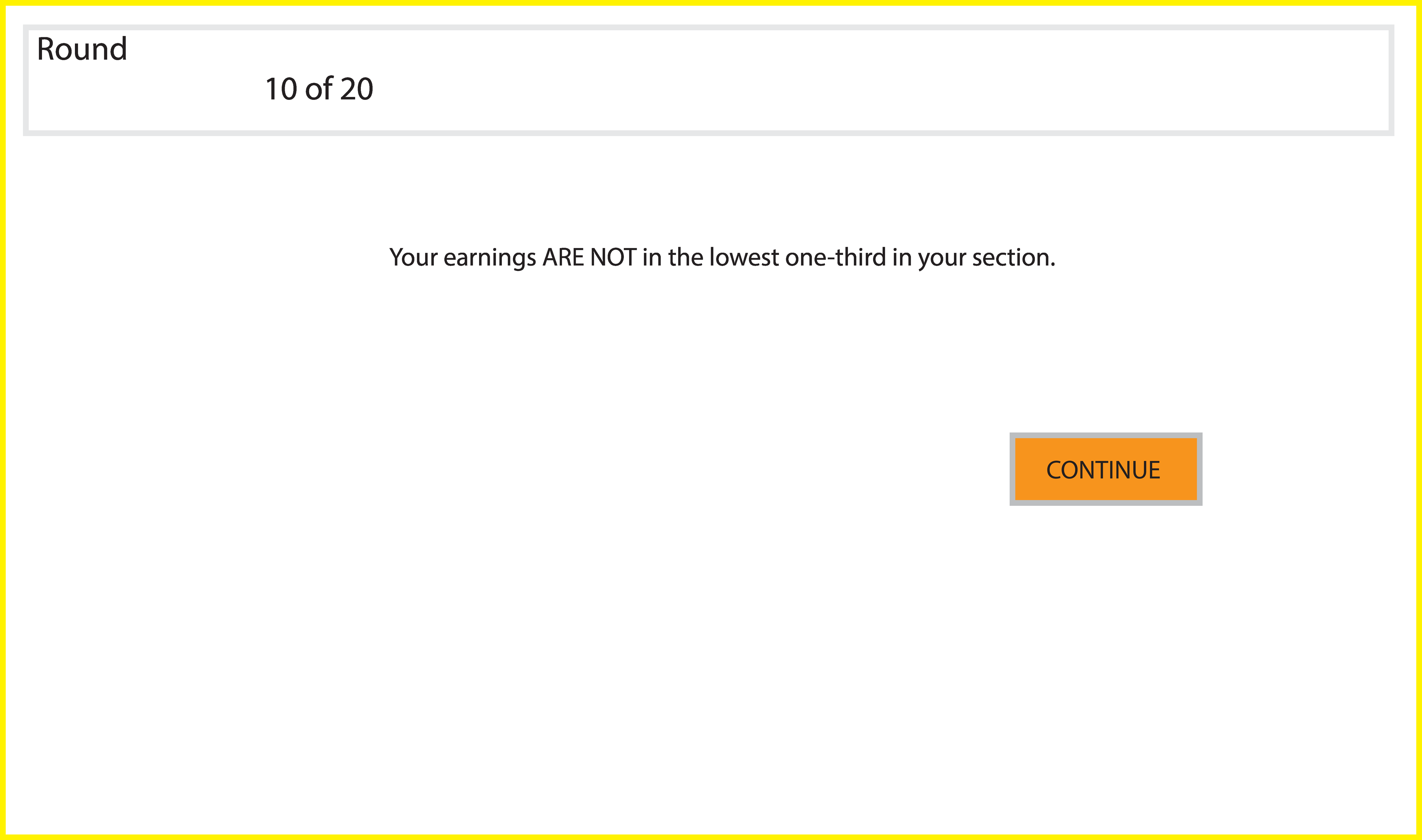

Supplement: S12 Fig — (TIF) [file pone.0157840.s012.tif]

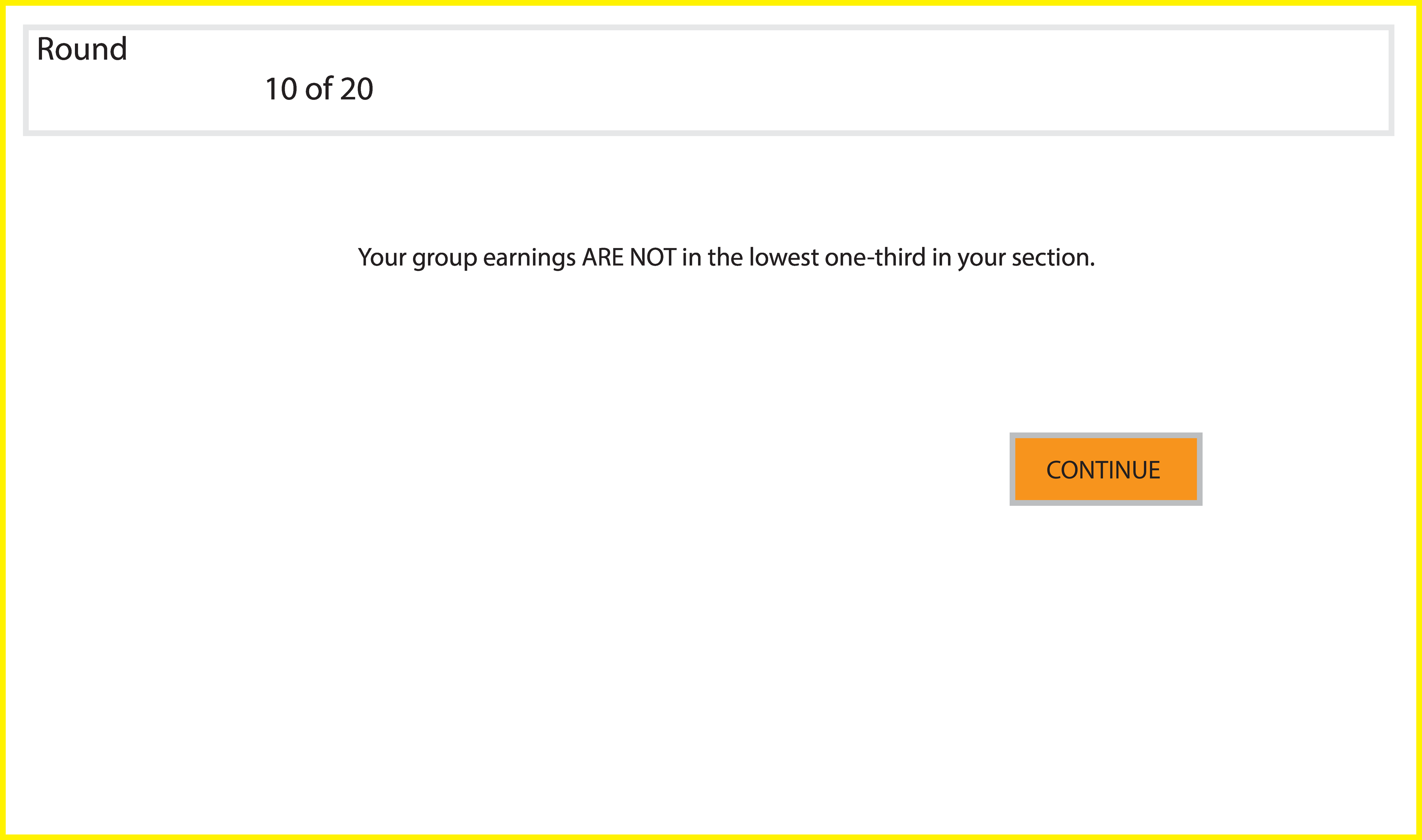

Supplement: S13 Fig — In both treatments subjects are given the same information. Only in the Group Extinction treatment do those groups exit the experiment. (TIF) [file pone.0157840.s013.tif]

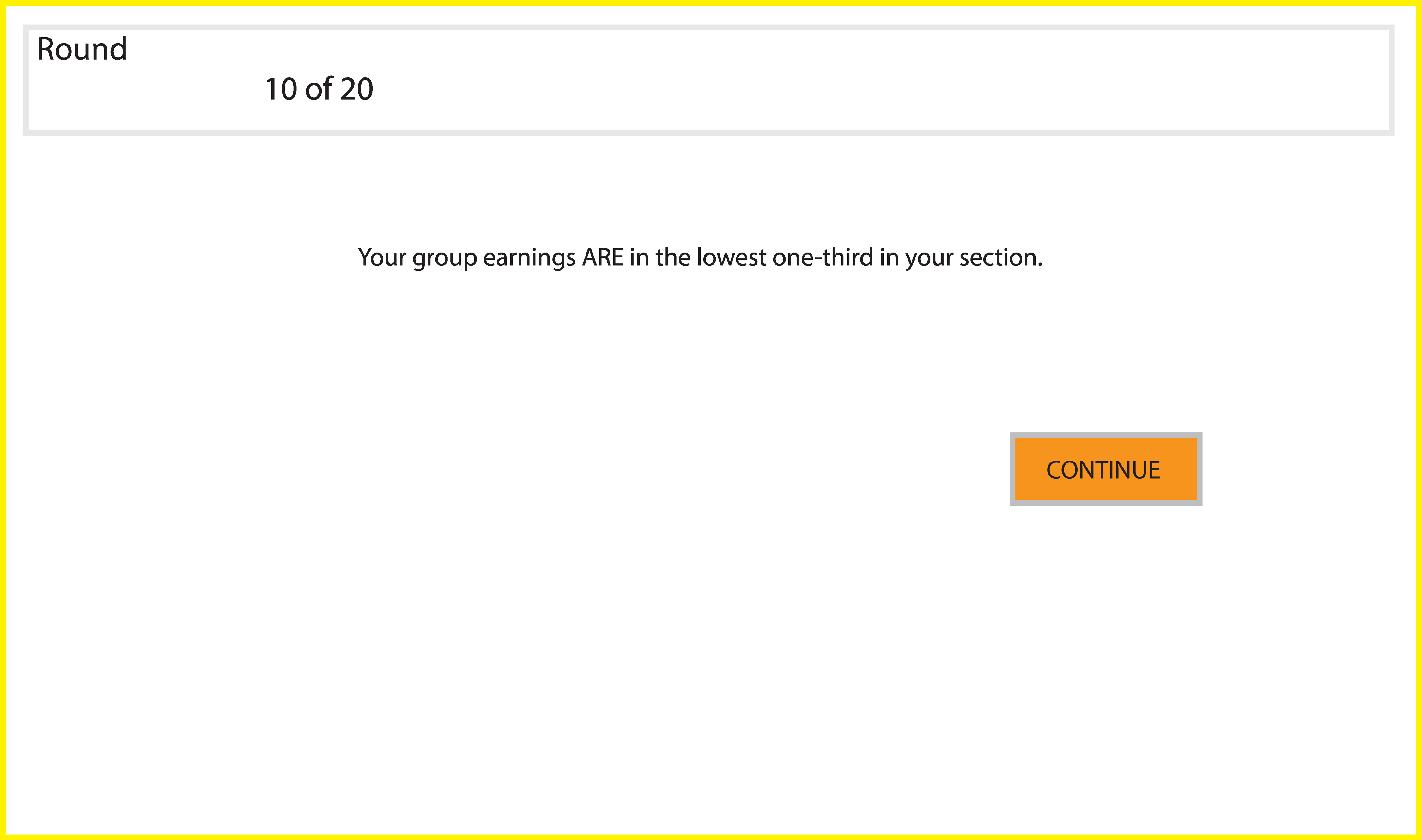

Supplement: S14 Fig — In both treatments subjects are given the same information. (TIF) [file pone.0157840.s014.tif]
