# Supplementary material for: Group-Level Selection Increases Cooperation in the Public Goods Game
Source: PLoS One. 2016 Aug 30;11(8):e0157840. doi: 10.1371/journal.pone.0157840 (PMC5004815; doi:10.1371/journal.pone.0157840)
Supplement: S1 File — (PDF) [file pone.0157840.s015.pdf]

Experimental Design

The Basic Public Goods Game

Linear public goods games, [1] have been the basic setting used to analyze cooperation in the presence of free rider incentives.

A standard linear public goods game identical to [2] serves as our baseline. In each period subjects are given a new endowment (50 monetary units – MUs) and they decide how to allocate their MUs to either a private account or to a group account. The private account returns at a rate of 1:1. All MUs placed in the group account are doubled by the experimenter and then shared equally by all members of the group. The group account rate of return is 1:2. The social dilemma is such that everyone is better off if everyone puts all of their MUs into the group account. However, each individual is better off free riding off the contributions of others, while keeping their private account assets.

Once allocation decisions are made, the information is collected and payoffs are calculated. After each period subjects are given information about individual payoffs, individual contributions to the group account as well as the overall contribution of their group.

Experimental Manipulations.

We use three manipulations of this standard public goods game. The first manipulation involves group comparison (GC). Subjects are told that at the end of 10 periods groups will be ranked by their total group performance (earnings). They will be told whether their group is in the top two-thirds or in the bottom one-third of group earnings in their section. Each section consists of 3 groups. During the second block, groups and sections remain the same. Subjects keep making decision under the same conditions that the first 10 periods, but they were told they would not receive group ranking information. The second manipulation involves group extinction (GE). Subjects were given the same instructions as in the GC manipulation, except they were told that groups in the bottom one-third of earnings would no longer participate in the same experiment. Instead they would be paid a fixed amount (their initial endowment) for the subsequent block of 10 periods. Subjects in groups that survived played the equivalent of the standard public goods game in the second block of 10 periods and received no feedback concerning group rankings. The third manipulation involves individual extinction (IE). Subjects were told that at the end of the first block of 10 periods that they would be told whether they were in the top two-thirds or in the bottom one-third of individual earnings in their section. Those in the bottom one-third would no longer participate in the same experiment and would earn a fixed amount in the subsequent block of 10 periods. Those that survived were randomly re-assigned to new groups and continued play in the second block of 10 periods and were not given any ranking information at the end of the second block.

Experimental procedures

This study includes data from experimental sessions run at the experimental laboratory at the University Of Valencia (LINEEX). Participants were recruited using LINEEX's own online recruiting software. All experimental sessions were conducted following the procedures established by LINEEX laboratory of the University of Valencia The ethics committee provided blanket approval for all economic-style experiments carried out in LINEEX that did not collect personal identifiers. Participants voluntarily registered in the electronic database participants' and gave their consent by accepting an invitation to attend an experimental session. Subjects were undergraduate students (mostly from business and economics) of the University of Valencia. We had a total of 196 participants (49 groups of four members).

The participants´ distribution across the treatments is summarized in Table S1: Baseline: a total of n=52; Group Comparison: n=48; Individual Extinction: n=48; Group Extinction: n=48. In the Baseline two sessions were run with seven and six groups, respectively. One session was run for each of the remaining treatments with 12 groups in each session.

All sessions used an identical protocol. Upon arrival, subject were welcomed and randomly seated at the visually separated computer terminals. Participants interacted anonymously via computer screens such that subjects did not know which of the other participants were in their group. Subjects were given a written set of instructions that the experimenter read aloud. The instructions included a quiz about how choices translate into earnings and about the composition of groups. Subjects had to answer all the questions correctly before the experiment could continue.

The experiment was computerized by using z-Tree [3]. Subjects knew that they would participate together for 10 periods. In the extinction treatments subjects knew that one-third of the subjects would not continue for another 10 periods. An average session lasted about 60 minutes. Subjects earned on average 17€, corresponding to the sum of the payoff earned in each round and the show-up fee.

1. Isaac RM, Walker JM, Thomas SH. Divergent Evidence on Free Riding: An Experimental Examination of Possible Explanations. Public Choice. 1984;43(4):113-49.
2. Croson R, Fatas E, Neugebauer T. Reciprocity, matching and conditional cooperation in two public goods games. Economics Letters. 2005;87(1):95-101.
3. Fischbacher U. z-Tree: Zurich toolbox for ready-made economic experiments. Experimental economics. 2007;10(2):171-8.
