# Supplementary material for: Group-Level Selection Increases Cooperation in the Public Goods Game
Source: PLoS One. 2016 Aug 30;11(8):e0157840. doi: 10.1371/journal.pone.0157840 (PMC5004815; doi:10.1371/journal.pone.0157840)
Supplement: S2 File — (PDF) [file pone.0157840.s016.pdf]

Experimental Instructions and Computer Screen Captures

Here we provide a translated copy of the experimental instructions we used in our group extinction experiments. The instructions for other treatments were adapted accordingly. The instructions were originally written in Spanish (by Enrique Fatas).

INSTRUCTIONS

The purpose of this experiment is to study how people make decisions in a particular situation. The instructions are simple and if you follow them carefully you will anonymously receive an amount of money at the end of the experiment, since none of you will be informed about others' earnings. Should you have a question, please raise your hand. Any communication with other participants is not permitted and will lead to an immediate exclusion from the experiment.

1. Just for participating in the experiment you receive a show-up fee of 300 MUs (Monetary Units).
2. The experiment consists of two blocks with 10 rounds in each block. In each round you will be part of a group of 4 participants and part of a section of 12 participants. Each section consists of 3 groups of 4 members.

BLOCK 1

3. The computer randomly determines the composition of your group and section at the beginning of the first block and does not change during the whole block. You will not know the identities of any of the section or group members.
4. At the beginning of each round, you will be endowed with 50 MUs (Monetary Units). Your only need to decide how many of your 50 MUs you want to assign to a Group Project (GP). The remainder will automatically be assigned to an Individual Project (IP).
5. Your earnings from the Individual Project equals to your assignment to the Individual Project and does not depend on the decisions of the other group members.
6. Your earnings from the Group Project depend on the total amount assigned to the Group Project (that is the sum of your assignment to the Group Project and the corresponding assignments of the other three members of your group). This total amount assigned to the Group Project will be multiplied by two and divided equally among the four group members.
7. Your round earnings are determined as follows:
   Individual earnings in each round = earnings from the Individual Project + earnings from the Group Project
   50 MU – assignment to Group Project+ (2 x Group Project)/4
8. After each round you will be informed of the individual assignments to the GP in your group, ranked from top to bottom, so you will not be able to identify decisions across periods. Additionally, you will be informed of your earnings in every round broken up to earnings from the Group Project and from the Individual Project. You will also receive all this information from all the previous rounds.

BLOCK 2

9. At the end of the first block of 10 rounds you will be informed of your group performance in your section. In particular, at the end of the round number 10 you will be informed of whether (or not) your group performance (group total earnings) over the first 10 rounds has been the lowest in your section.
10. Whether (or not) your group performance in the first block has been the lowest in your section will have consequences. At the beginning of the second block the group with the lowest performance (in each section) will become "group B". The other 2 groups will become "group A".
11. Group A members will face the same decisions that they did during the first block of 10 rounds. That means you only need to decide how many of your 50 MU you want to assign to a Group Project (GP). The remainder will automatically be assigned to an Individual Project (IP).
12. Your round earnings is determined as follows:

Individual earnings = earnings from the Individual Project + earnings from the Group Project

50 MU – assignment to Group Project+ (2 x Group Project)/4Group B

13. Group B members stop participating in the experiment. They will receive the initial endowment of 50 MUs each round. They will remain seated and facing the same decisions that group A members, however these decisions will NOT have any consequences on their earnings.
14. At the end of the experiment, the sum of your individual earnings over the 20 rounds will be privately paid to you in cash at the exchange rate of 100 MU=1€

Quiz

1) My earnings depend on actions from participants from different groups.
☐ True ☐ False

2) My earnings in each round do not depend on decisions from previous rounds
☐ True ☐ False

3) Group composition remains the same thought the experiment
☐ True ☐ False

4) Please fill the following table.

Group Member	Assignment to the GP	Assignment to the IP	GP	Payoffs from IP	Payoffs from GP
1	0				
2	25				
3	75				
4	100				

5) Earnings depend on information provided after the first block of the experiment.
☐ True ☐ False
