# Supplementary material for: Group-Level Selection Increases Cooperation in the Public Goods Game
Source: PLoS One. 2016 Aug 30;11(8):e0157840. doi: 10.1371/journal.pone.0157840 (PMC5004815; doi:10.1371/journal.pone.0157840)
Supplement: S1 Table — (PDF) [file pone.0157840.s017.pdf]

S1 Table. Summary of treatments.

	Treatments			
	Comparison	Extinction	# Groups	# Subjects
Baseline	No	No	13	52
Group Comparison	Yes	No	12	48
Individual Extinction	No	Yes	12/8	48/32
Group Extinction	Yes	Yes	12/8	48/32
