# Supplementary material for: Group-Level Selection Increases Cooperation in the Public Goods Game
Source: PLoS One. 2016 Aug 30;11(8):e0157840. doi: 10.1371/journal.pone.0157840 (PMC5004815; doi:10.1371/journal.pone.0157840)
Supplement: S2 Table — Means are pooled over all periods and then broken out by the first and second blocks. (PDF) [file pone.0157840.s018.pdf]

S2 Table. Means (standard deviations) of contributions at the group level. Means are pooled over all periods and then broken out by the first and second blocks.

		All 20 periods		First Block		Second Block	
	**Obs**	**Contributions (MUs)**	**Earnings (MUs)**	**Contributions (MUs)**	**Earnings (MUs)**	**Contributions (MUs)**	**Earnings (MUs)**
**Baseline**	13	60.50	1302.52	70.88	677.21	50.12	625.31
		(33.25)	(118.54)	(34.45)	(77.08)	(28.47)	(59.56)
**Group Comparison**	12	76.72	1383.58	83.88	709.69	69.56	673.90
		(48.78)	(209.37)	(47.38)	(119.31)	(49.16)	(119.31)
**Individual Extinction**	12	62.56	1296.79	73	682.5	46.89	617.22
		(42.41)	(176.77)	(43.29)	(118.49)	(35.77)	(85.18)
**Group Extinction**	12	159.73	1711.8	184.13	960.31	123.14	807.84
		(55.99)	(207.42)	(25.91)	(52.20)	(67.87)	(147.71)
