# Supplementary material for: Group-Level Selection Increases Cooperation in the Public Goods Game
Source: PLoS One. 2016 Aug 30;11(8):e0157840. doi: 10.1371/journal.pone.0157840 (PMC5004815; doi:10.1371/journal.pone.0157840)
Supplement: S3 Table — The comparisons are for all 20 periods, the first block and the second block. Each comparison includes contributions and earnings information. In the table BSL = Baseline; GC = Group Comparison; IE = Individual Extinction; and GE = Group Extinction. (PDF) [file pone.0157840.s019.pdf]

S3 Table. **P-values for Wilcoxon Mann-Whitney tests for pairwise treatment differences.** The comparisons are for all 20 periods, the first block and the second block. Each comparison includes contributions and earnings information. In the table BSL=Baseline; GC=Group Comparison; IE=Individual Extinction; and GE=Group Extinction.

Group Contributions (MUs)

All 20 periods

	**BSL**	**GC**	**IE**
**GC**	0.2011	-	-
**IE**	0.7682	0.1195	-
**GE**	0.0001	0.0001	0.0001

Group Earnings (MUs)

All 20 periods

	**BSL**	**GC**	**IE**
**GC**	0.1826	-	-
**IE**	0.7965	0.0942	-
**GE**	0.0001	0.0022	0.0001

$N_{BSL} = 13$, $N_{GC} = 12$, $N_{IE} = 12$, $N_{GE} = 12$.

Group Contributions (MUs)

First Block

	BSL	GC	IE
**GC**	0.2314	-	-
**IE**	0.9134	0.3865	
**GE**	0.0001	0.0001	0.0001

Group Earnings (MUs)

First Block

	BSL	GC	IE
**GC**	0.2534	-	-
**IE**	0.9566	0.2987	-
**GE**	0.0001	0.0001	0.0001

$N_{BSL} = 13$, $N_{GC} = 12$, $N_{IE} = 12$, $N_{GE} = 12$.

Group Contributions (MUs)

Second Block

	BSL	GC	IE
**GC**	0.1346	-	-
**IE**	0.8563	0.2170	-
**GE**	0.0018	0.0308	0.0033

Group Earnings (MUs)

Second Block

	BSL	GC	IE
**GC**	0.1210	-	-
**IE**	0.5145	0.2170	-
**GE**	0.0018	0.0308	0.0033
