# Supplementary material for: Group-Level Selection Increases Cooperation in the Public Goods Game
Source: PLoS One. 2016 Aug 30;11(8):e0157840. doi: 10.1371/journal.pone.0157840 (PMC5004815; doi:10.1371/journal.pone.0157840)
Supplement: S4 Table — Analysis is across all periods and then by first and second block. Baseline treatment is the omitted category. (PDF) [file pone.0157840.s020.pdf]

S4 Table. Individual Level Regressions (Panel Random-effects generalized least squares). Analysis is across all periods and then by first and second block. Baseline treatment is the omitted category.

	All 20 periods	First Block	Second Block
Group Comparison (GC)	4.053	3.248	4.859
	(0.096)	(0.266)	(0.067)
Individual Extinction (IE)	1.479	0.529	2.430
	(0.655)	(0.868)	(0.298)
Group Extinction (GC)	21.96***	28.31***	15.61***
	(0.000)	(0.000)	(0.000)
Constant	21.45***	20.32***	19.64***
	(0.000)	(0.000)	(0.000)
Observations	3920	1960	1960
Post regression tests:			
GC = IE:	p = 0.4788	p=0.4269	p=0.4199
GC= GE	p=0.0000	p=0.0000	p=0.0147
IE = GE	p=0.0000	p=0.0000	p=0.0019

p-values in parentheses

* $p < 0.05$, ** $p < 0.01$, *** $p < 0.001$

Dep. variable: Individual contribution in each period. Std. errors clustered on independent groups. Includes period dummies (not reported).
