# Supplementary material for: Group-Level Selection Increases Cooperation in the Public Goods Game
Source: PLoS One. 2016 Aug 30;11(8):e0157840. doi: 10.1371/journal.pone.0157840 (PMC5004815; doi:10.1371/journal.pone.0157840)
Supplement: S5 Table — An additional control for the period is included. Baseline treatment is the omitted category. (PDF) [file pone.0157840.s021.pdf]

S5 Table. Individual Level Regressions (Panel Random-effects generalized least squares). An additional control for the period is included. Baseline treatment is the omitted category.

	All 20 periods	First Block	Second Block
Period	-0.893***	-0.894***	- 1.794***
	(0.000)	(0.000)	(0.000)
Group Comparison (GC)	4.053	3.248	4.859*
	(0.095)	(0.265)	(0.067)
Individual Extinction (IE)	1.479	0.529	2.430
	(0.671)	(0.867)	(0.297)
Group Extinction (GE)	21.96***	28.31***	15.607***
	(0.000)	(0.000)	(0.000)
Constant	24.50***	22.64***	40.333***
	(0.000)	(0.000)	(0.000)
Observations	3920	1960	1960
Post regression tests:			
GC = IE:	p=0.4967	p=0.4259	p=0.4190
GC= GE	p=0.0000	p=0.0000	p=0.0145
IE = GE	p=0.0000	p=0.0000	p=0.0017

p-values in parentheses

* $p < 0.05$, ** $p < 0.01$, *** $p < 0.001$

Dep. variable: Individual contribution in each period. Std. errors clustered on independent groups.
