# Supplementary material for: Group-Level Selection Increases Cooperation in the Public Goods Game
Source: PLoS One. 2016 Aug 30;11(8):e0157840. doi: 10.1371/journal.pone.0157840 (PMC5004815; doi:10.1371/journal.pone.0157840)
Supplement: S6 Table — The first part of the table indicates the total average MUs given by the group (out of a possible 200 MUs that could be given in each period). The second part of the table presents Wilcoxon rank-sum tests between the Baseline treatments. (PDF) [file pone.0157840.s022.pdf]

S6 Table. Averages by groups across periods for the baseline treatment. The first part of the table indicates the total average MUs given by the group (out of a possible 200 MUs that could be given in each period). The second part of the table presents Wilcoxon rank-sum tests between the Baseline treatments.

Mean Group Contributions (SD).

	Obs	All 20 periods	First Block	Second Block
Baseline 10+ 10	6	61.33	63.75	58.9
		(36.85)	(40.77)	(32.63)
Baseline 20	7	59.80	77.00	42.6
		(30.04)	(27.00)	(22.11)

Wilcoxon rank-sum tests for differences between Baseline treatments

	All 20 periods	First Block	Second Block
	Baseline 10+10	Baseline 10+10	Baseline 10+10
Baseline 20	z = 0.429<br>p = 0.6682	z = 1.429<br>p = 0.1531	z = -1.286<br>p = 0.1985
